# Identification of DLL3-related genes affecting the prognosis of patients with colon adenocarcinoma

**DOI:** 10.3389/fgene.2023.1098190

**Published:** 2023-05-18

**Authors:** Jinyu Xiang, Wenjing Gong, Jiannan Liu, Huijuan Zhang, Ming Li, Rujian Wang, Yaodong Lv, Ping Sun

**Affiliations:** ^1^ Departments of Oncology, Yantai Yuhuangding Hospital, Shandong University, Yantai, Shandong, China; ^2^ Departments of Neurology, Yantai Yuhuangding Hospital, Shandong University, Yantai, Shandong, China

**Keywords:** DLL3, colon adenocarcinoma, AMPK signaling pathway, mitophagy, prognosis

## Abstract

**Background:** Delta-like ligand 3 (DLL3) is one of the NOTCH family of ligands, which plays a pro- or anti-carcinogenic role in some cancers. But the role of DLL3 in colon adenocarcinoma (COAD) has not been studied in depth.

**Materials and methods:** First, we used Kaplan-Meier (K-M) curve to evaluate the effect of DLL3 on the prognosis of COAD in The Cancer Genome Atlas (TCGA), which was further validated in clinical samples for immunohistochemistry. Then we screened for differentially expressed genes (DEGs) of DLL3 by analyzing datasets of COAD samples from Gene Expression Omnibus (GEO) and TCGA. Gene Ontology (GO), Kyoto Encyclopedia of Genes and Genomes (KEGG) analyses, and Gene Set Enrichment Analysis (GSEA) were conducted to explore the underlying mechanisms of DLL3-related in the development and prognosis of COAD. On the basis of DLL3-related signature genes, a prognostic model and a nomogram were constructed. Finally, CIBERSORT was applied to assess the proportion of immune cell types in COAD sample.

**Results:** Survival analysis showed a significant difference in overall survival between high- and low-expression group (*p* = 0.0092), with COAD patients in the high-group having poorer 5-year survival rate. Gene functional enrichment analysis revealed that DLL3-related DEGs were mainly enriched in tumor- and immunity-related signaling pathways, containing AMPK pathway and mitophagy-animal. The comparison of COAD tumor and normal, DLL3 high- and low-expression groups by GSEA found that AMPK signaling pathway and mitophagy-animal were inhibited. Nomogram constructed from DLL3-related signature genes had a good predictive effect on the prognosis of COAD. We found the highest correlation between DLL3 and interstitial dendritic cell (iDC), natural killer (NK) cell and Interstitial dendritic cell (Tem). DLL3 was also revealed to be diagnostic for COAD. In clinical sample, we identified higher DLL3 expression in colon cancer tissue than in adjacent control (*p* < 0.0001) and in metastasis than in primary lesion (*p* = 0.0056). DLL3 expression was associated with stage and high DLL3 expression was observed to predict poorer overall survival (*p* = 0.004).

**Conclusion:** It suggested that DLL3 may offer prognostic value and therapeutic potential for individualized treatment of COAD, and that it may has a diagnostic role in COAD.

## 1 Introduction

According to latest statistics, the number of new colon cancer cases worldwide in 2020 was 1,148,515, the fifth highest incidence rate, and also the fifth highest mortality rate with 576,858 deaths (Sung et al., 2021). Colon adenocarcinoma (COAD) is the most predominant pathological type of colon cancer. The main treatment options for COAD include endoscopic resection, surgery, radiotherapy, chemotherapy, targeted therapy and immunotherapy, among others (Dekker et al., 2019). The choice of treatment is based on the stage of the disease, the patient’s physical condition and in particular molecular typing of tumor, which is determined after multidisciplinary discussion (Stintzing, 2014). Despite some advances in diagnosis and treatment, patients with COAD frequently develop recurrence and metastasis, which greatly reduces the 5-year survival rate (Biller and Schrag, 2021). Therefore, there is an urgent need to explore the molecular mechanisms underlying the development of COAD and to search for potential biomarkers to improve the diagnosis, treatment and prognosis of COAD.

DLL3 (Delta-like Ligand 3) is a member of the Delta/Serrate/Lag2 (DSL) Notch receptor ligands, which in mammals include the five ligands DLL1, DLL3, DLL4, JAG1, and JAG2 (D'Souza et al., 2008). Different ligands act through different mechanisms. The ligands DLL1, DLL4, JAG1, and JAG2 each activate Notch receptor signaling, as DLL1 and DLL4 are involved in tumor angiogenesis. In contrast, DLL3 is mainly located in the Golgi apparatus and binds to Notch receptors only through cis interactions, thereby obtaining a specific inhibitory and cascade modulatory effect (Xiu et al., 2020). DLL3 plays a crucial role in Notch signaling, influencing a variety of cellular processes including differentiation, proliferation, survival and apoptosis (Bulman et al., 2000; Hu et al., 2018). In recent years, studies have identified high expression of DLL3 (about 80%) in small-cell lung cancer (SCLC) and other high-grade neuroendocrine tumors (Saunders et al., 2015), with low expression in normal tissues, which provides potential for targeted therapies. A review of related articles found that DLL3 is highly expressed in neuroendocrine-related tumors (Fujiwara et al., 1993; Matsuo et al., 2019). Notably, overexpression of DLL3 was associated with shorter overall survival (OS) in endometrial cancer (Wang et al., 2018), ovarian cancer (Jia et al., 2019), breast cancer (Yuan et al., 2021) and small-cell bladder cancer (Koshkin et al., 2019). The safety of antibody-drug conjugate (ADC) drugs (Uprety et al., 2021) and the refractory nature of SCLC have led to less satisfactory clinical trials of drugs targeting DLL3 in SCLC (Johnson et al., 2021; Mansfield et al., 2021; Matsuo et al., 2021), there are still different drug forms being actively explored. The ligand DLL3 bound to the Notch receptor and acts an inhibitor of Notch pathway (Chapman et al., 2011). Depending on the tumor type and cell growth environment, activation of DLL3 can play a pro- or anti-cancer role. DLL3 has been described to exhibit pro-carcinogenic action in small cell lung cancer (Sabari et al., 2017; Vitorino et al., 2021), breast cancer (Ayyanan et al., 2006; Yuan et al., 2021), pituitary tumor (Wang et al., 2017) and acute myeloid leukaemia (Yan et al., 2010), but an anti-cancer effect in hepatocellular carcinoma (Maemura et al., 2013), and glioma (Turchi et al., 2013; Jungk et al., 2016). NOTCH signaling is associated with tumor formation, self-renewal and expression of secretory cell lineage differentiation in colon cancer (Sikandar et al., 2010). It has also been reported that Notch is dysregulation in colon cancer. Notch receptors and ligands are both believed to be involved in the more aggressive phenotype of colon cancer, especially Notch-1, Notch-3, Jagged-1 and DLL4 (Reedijk et al., 2008) (Supplementary Figure S1). The role of other receptors and ligands on the notch pathway in colon cancer has been studied previously (Reedijk et al., 2008), but in terms of DLL3’s role in COAD development, little is known (Pu et al., 2021). It is therefore worth exploring the mechanism of action of DLL3 in COAD and whether it can be a therapeutic target.

In this study, we analyzed the differentially expressed genes (DEGs) between DLL3 high- and low-expression group in The Cancer Genome Atlas (TCGA) and Gene Expression Omnibus (GEO) database. Furthermore, Gene Ontology (GO), Kyoto Encyclopedia of Genes and Genomes (KEGG) and Gene set enrichment analysis (GSEA) were applied to the enrichment analysis of the molecular function and pathway associated with DLL3-related intersection genes or DLL3. The aim of this study was to identify the impact of DLL3-related genes on the development and prognosis of COAD. We also collected clinical samples to verify the expression of DLL3 at protein level and to explore its clinical significance in COAD.

## 2 Materials and methods

### 2.1 Data collection and processing

The gene expression profile data of COAD were obtained from three public datasets, including TCGA database and two independent datasets retrieved from GEO (GSE39582 (Marisa et al., 2013) based on platform GPL570 and GSE44076 (Sole et al., 2014) based on platform GPL13667). According to the COAD information recorded by the TCGA, 478 tumor tissue and 41 healthy control tissue samples were obtained. The GSE39582 dataset included 566 tumor and 19 non-tumor colorectal mucosal tissue samples. The GSE44076 dataset included 98 colon tumors and their adjacent pairs of 98 normal para-cancer mucosal samples (The 50 healthy colon mucosa cases in this gene expression profile were not included in study because of the analysis in colon tissue.). Guidelines and regulations of the above-mentioned databases were followed in the analyses carried out in our study. Supplementary Figure S2 illustrated the flow chart of the overall study design.

### 2.2 Differentially expressed gene screening

To explore DLL3-related genes that may affect the prognosis of COAD, we performed an in-depth analysis of the above dataset. Firstly, the expression spectrum of DLL3 was used to take the optimal cutoff value of the continuous variable based on the Kaplan–Meier (K-M) curve (Ranstam and Cook, 2017) to sort COAD samples according to their expression levels in TCGA data. In addition, differential gene expression analysis between COAD and control samples or between high- and low-expression group in TCGA using the “DESeq2” R package (Love et al., 2014) yielded DEGs (*p* < 0.05). In the GSE39582 and GSE44076 datasets, DEGs were obtained by differential gene expression analysis of COAD and control samples using the “limma” R package (*p* < 0.05) (Ritchie et al., 2015). Ultimately, DLL3-related intersection genes with the same differential expression direction were screened out in the four groups of DEGs.

### 2.3 Enrichment analyses

To investigate the function and pathway associated with DLL3, we performed GO and KEGG enrichment analysis, intersection genes was involved and “clusterProfiler” R package (Yu et al., 2012) was applied in the analysis (*p* < 0.05). Gene Set Enrichment Analysis (GSEA) was conducted for DLL3 high-vs. low-expression group and COAD tumor vs. normal in TCGA expression profile.

### 2.4 Univariate cox regression analysis

The intersection genes were subjected to univariate Cox regression analysis to identify the gene that significantly affected the prognosis of COAD patients. The area under curve (AUC) values of these genes in TCGA and GSE39582 dataset were calculated using the “pROC” R package (Robin et al., 2011), respectively. The “RandomSurvivalForest” R package (Wang and Zhou, 2017) employed to rank the genes based on their relative importance. Least absolute shrinkage and selection operator (LASSO) Cox regression models were then constructed for the signature genes.

### 2.5 Construction and validation of risk score model and nomogram

Multivariate Cox regression analysis was performed based on signature genes, and risk scores were calculated. The effect of signature genes on the prognosis of COAD was evaluated by using the “rms” R package (Xu et al., 2021) to construct a nomogram (Iasonos et al., 2008). Hazard ratios (HR) for the signature genes were determined using the “forestplot” R package (Fang et al., 2021). Gene signatures with HR > 1 were identified as prognostic risk factors, while genes with HR < 1 were considered protective.

### 2.6 Evaluation of immune cell infiltration

In order to explore the relevance of DLL3 to immune cells in COAD, the Cell type Identification by Estimating Relative Subsets of RNA Transcripts (CIBERSORT) algorithm was implemented to assess the proportion of immune cell types in the COAD samples. A single sample gene set enrichment analysis (ssGSEA) in the GSVA R package was used to calculate the level of immune cell infiltration in COAD and control samples. The “limma” R package was used to calculate the difference in immune cell infiltration between COAD and healthy control samples or between DLL3 high-group and low-group.

### 2.7 Tumor samples

We retrospectively collected 104 pairs of primary tumors and adjacent non-tumor tissue specimens surgically resected from patients with colon cancer in Yantai Yuhuangding Hospital, Shandong University, Shandong, China from 2014 to 2016. In 24 patients we obtained puncture specimens of metastatic lesions, which were paired with the primary lesions for immunofluorescence analysis. All specimens were routinely fixed in 10% formalin, paraffin embedded and stored at room temperature. Neither radiotherapy nor chemotherapy had been administered prior to surgery. Written informed consent was obtained from all patients and all procedures were approved by Ethics Committee of Yantai Yuhuangding Hospital (The number of ethic approval is 2022–226).

### 2.8 Immunofluorescence analysis

To determine whether DLL3 has an important role in COAD, its levels were measured by immunofluorescence analysis in colon tissue with a specific monoclonal antibody raised against human DLL3. For immunofluorescence analysis, tissue sections were fixed in 4% formaldehyde for 15 min at room temperature. Afterwards, cell membranes were permeabilized with 0.3% Triton X-100 for 15 min, and then blocked with 1% bovine serum albumin for 30 min at room temperature. Specific primary (DLL3, abcam, ab229902, 1:100; overnight incubation at 4°C) and secondary (Goat Anti-Rabbit IgG H&L (Alexa Fluor^®^ 488); Abcam, ab150077; 1:200; room temperature for 90 min) antibodies were used. Nuclei were stained with Hoechst 33,342. Each step was followed by three 5-min washes in phosphate-buffered saline. Each slide was scored blindly by two pathologists under ×400 magnification according to the manufacturer’s recommended criteria. The immunofluorescence staining was interpreted in a semiquantitative manner and scored as follows: 1+, 2+, 3+, 4+, and 5+. Intensity scores of 1+ or 2+ were designated as low-expression and 3+, 4+, 5+ were designated as high-expression.

### 2.9 Statistical analysis

Fisher’s exact test or chi-square test was used to determine the correlation between DLL3 and clinical characteristics. DLL3 expression in colon cancer and adjacent control, primary and metastatic lesion was compared by pair *t*-test. In addition, the correlation between DLL3 and overall survival in COAD was explored by K-M curves and univariate/multifactor Cox proportional risk regression analysis. We used Spearman correlation to explore the associations between variables. All bioinformatics analyses in this study were performed based on the Bioinforcloud platform (http://www.bioinforcloud.org.cn), and experiment statistical analyses were conducted using SPSS software (version 26.0). *p* < 0.05 was considered to be statistically significant.

## 3 Results

### 3.1 Differentially expressed gene screening

**FIGURE 1:**
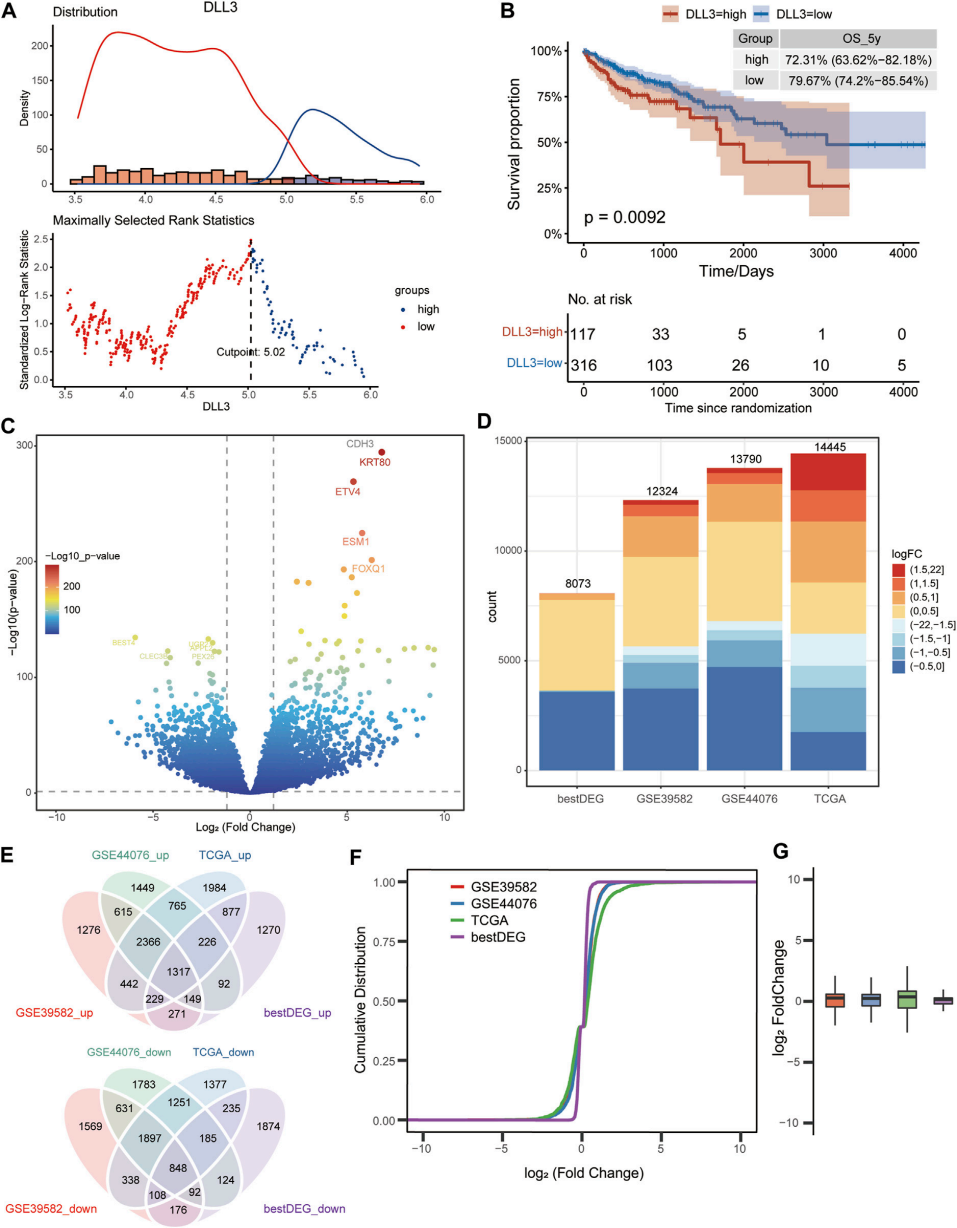
Differentially expressed genes between colon adenocarcinoma (COAD) and control. **(A)** COAD samples from TCGA data were divided into high- and low-expression group according to the best cutoff value of DLL3 expression. **(B)** K-M curves demonstrating the difference in overall survival probability of COAD patients between high- and low-expression group. **(C)** Volcano plot of DEGs between COAD and control in TCGA data. **(D)** Statistical histogram of the four groups of DEGs. **(E)** Intersection of the four groups with up- or downregulated DEGs. (The bestDEG_up refers to the upregulated genes in the 8,073 differentially expressed genes.) **(F)** Cumulative distribution plot and the log2 fold change plot **(G)** showing the similarity of intersecting gene expressions.

The COAD samples in TCGA were divided into high- and low-expression groups according to DLL3 expression (Figure 1A). A significant difference in OS was found between the high- and low-expression groups, with COAD patients in the high-expression group having poorer OS rates (Figure 1B). Differential gene expression analysis between COAD and control samples in TCGA data, GSE39582 dataset and GSE44076 dataset identified 14,452, 12,324, and 13,790 DEGs (Figures 1C, D), respectively. Analysis of variance between DLL3 high-group and low-group obtained 8,073 DEGs (bestDEG) (*p* < 0.05). Ultimately, 1,371 upregulated and 848 downregulated intersection genes with the same differential expression direction were screened out in the above four groups of DEGs (Figure 1E). Among these four groups, TCGA, GSE39582 and GSE44076 had higher similarity in the fold change of differential expression of the intersecting genes (Figure 1F, G).

### 3.2 GO terms and KEGG enrichment analysis

**FIGURE 2:**
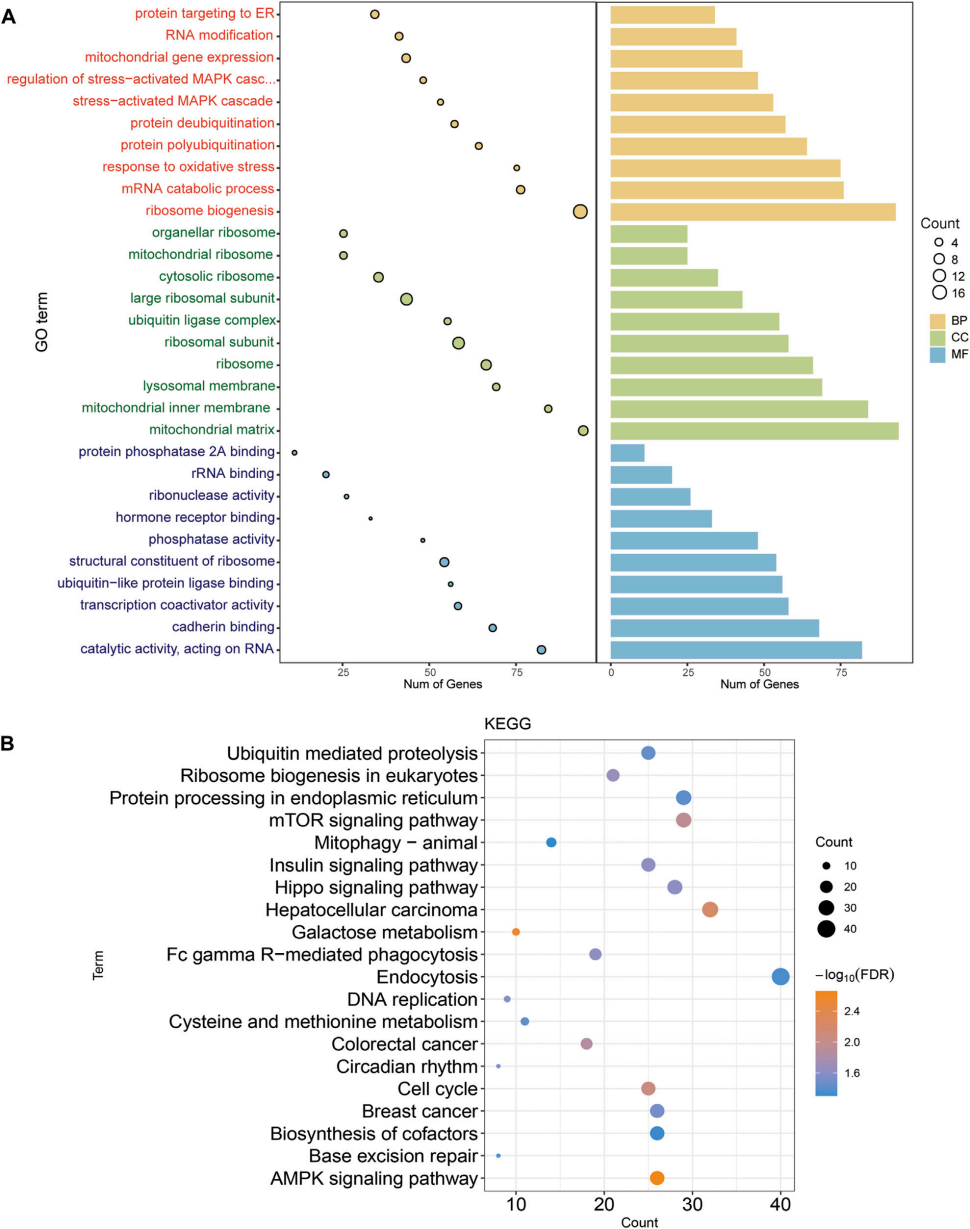
Enrichment analysis of intersection genes. **(A)** The main GO function of intersecting gene enrichment. The larger the circle, the more genes were involved in this function. **(B)** Major KEGG pathway for intersection gene enrichment. Colour represent *p*-value. The larger the circle, the more genes were involved in this function.

The GO results (Figure 2A) include biological processes (BP), cell composition (CC) and molecular functions (MF). The BP of intersection gene enrichment mainly involved ribosome biosynthesis, mitochondrial gene expression, proteins targeting ER, and MAPK cascade regulating stress activation. CC mainly involved ribosomal subunits, mitochondrial matrix, large ribosomal subunits, and ribosomes. MF mainly concerned the structural components of ribosomes, ubiquitin-like protein ligase binding, catalytic activity acting on RNA, and transcriptional co-activator activity. Among the KEGG enrichment results, the AMPK signaling pathway, cell cycle, mTOR signaling pathway, mitophagy−animal, hepatocellular carcinoma, colorectal cancer, breast cancer were mainly involved (Figure 2B). The above analysis indicates that DLL3-related genes are mainly involved in cancer-related and immune-related signaling pathways, thus suggesting that DLL3 is significantly involved in the development of tumor.

### 3.3 Gene set enrichment analysis

**FIGURE 3:**
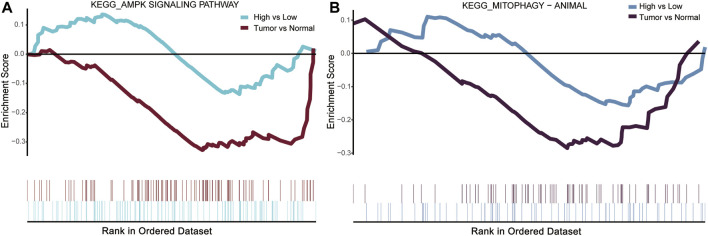
Gene set enrichment analysis (GSEA) results for DLL3 high-vs. low-expression group and COAD tumor vs. normal in TCGA. **(A)** AMPK signaling pathway was suppressed in DLL3 high-expression group and tumor group. **(B)** Mitophagy-animal was suppressed in DLL3 high-expression group and tumor group.

The previous KEGG enrichment analysis identified AMPK and mitophagy-animal among the pathways involved in the intersection genes. The AMPK pathway activates mitophagy which is well established at present (Herzig and Shaw, 2018). Gene set enrichment analysis (GSEA) was performed for DLL3 high-vs. low-expression group and COAD tumor vs. normal in TCGA expression profile. Comparison both revealed that the AMPK signaling pathway and mitophagy-animal were inhibited (Figures 3A, B).

### 3.4 Univariate cox regression analysis

**FIGURE 4:**
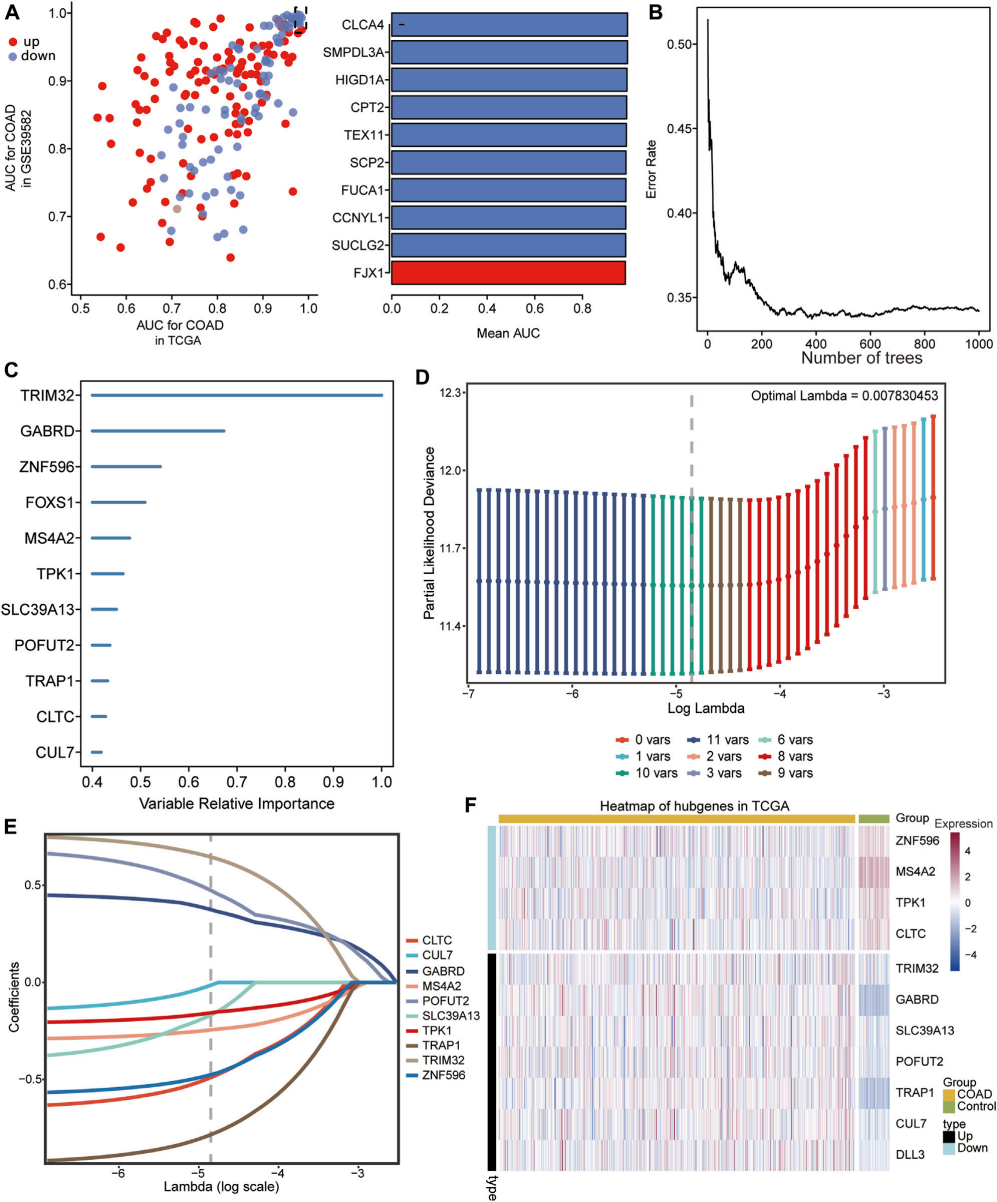
Screening of characteristic genes. **(A)** Area under curve (AUC) value of 199 genes significantly affecting prognosis of COAD patients in TCGA and GSE39582 data (left), and the top 10 genes with the largest AUC values in TCGA and GSE39582 data (right). Upregulated expression is shown in red, and downregulated expression is shown in blue. **(B)** Relationship between error rate and number of classification trees. **(C)** Importance ranking of the 11 feature genes. **(D)** The choice of adjustment parameter λ in the least absolute shrinkage and selection operator (LASSO) model was validated by minimum criteria using 10-fold crossover. **(E)** LASSO coefficient spectra of 10 feature genes. **(F)** Heat map of expression of the 10 signature genes in COAD and control samples.

The intersection genes were subjected to univariate cox regression analysis, 199 genes that significantly affected the prognosis of COAD patients were identified, then the AUC values of these genes in TCGA and GSE39582 dataset were calculated (Figure 4A), and 162 genes with AUC >0.7 were screened for sequencing of relative importance. The genes with correlation >0.4 were identified as final signature genes (Figure 4B). Eventually, we identified 11 signature genes, and Figure 4C showed the order of relative importance of top 11 genes. Then a LASSO Cox regression model was constructed with 11 signature genes. The optimal value of lambda (λ = 0.007830453) was adjusted by tenfold cross validation to give the minimum mean cross validation error (Figure 4D). Ten signature genes with non-zero coefficients were finally screened (Figure 4E). Subsequently, the expression of 10 signature genes in COAD and control samples from TCGA data was demonstrated using heatmap (Figure 4F).

### 3.5 Construction and validation of risk score model and nomogram

**FIGURE 5:**
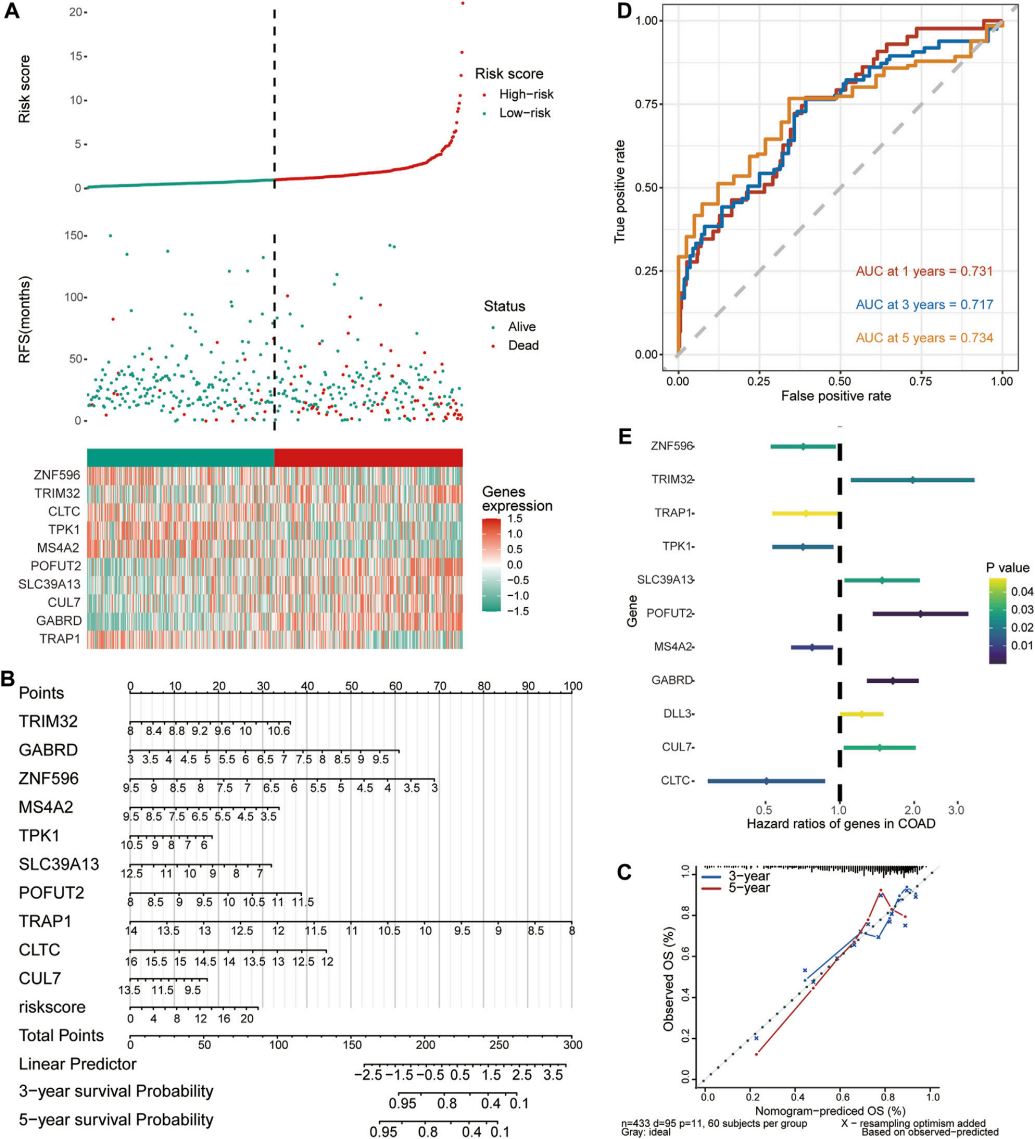
Predictive evaluation of characteristic genes for prognosis of COAD patients. **(A)** Risk score model, patient survival status and expression of 10 signature genes in TCGA data. **(B)** A column line graph was created to visualize the effect of the expression of the signature genes and the risk score model on patient prognosis. **(C)** Corrected curves of 3-year and 5-year OS of COAD patients in the TCGA dataset. **(D)** Time-dependent ROC curves for median risk scores. **(E)** Cox regression analysis of the signature gene and DLL3.

Multivariate Cox regression analysis was performed based on 10 signature genes, and risk scores were calculated. According to the median risk score, a risk score model was constructed to divide COAD samples into high-risk and low-risk groups (Figure 5A). The effect of signature genes on the prognosis of COAD was evaluated to construct a Nomogram (Figure 5B). Among them, TRAP1 contributed the most to prognosis prediction of patients. In addition, the calibration curves for the 3-year and 5-year OS shown in Figure 5C showed that the Nomogram model has a better prediction of prognosis. Time-dependent ROC curves revealed that median risk score predicted 1,3,5-year OS for COAD patients with AUC >0.7 (Figure 5D). The forest plot shows the risk ratios for the signature genes. Gene signatures with HR > 1 were identified as prognostic risk factors, while genes with HR < 1 were considered protective (Figure 5E).

### 3.6 Evaluation of immune cell infiltration

**FIGURE 6:**
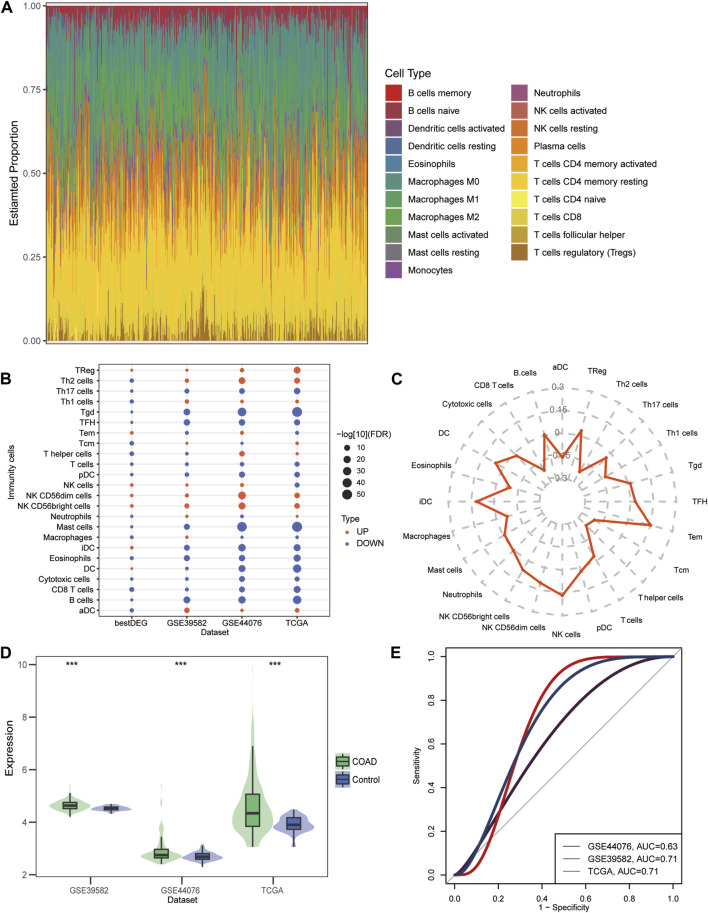
Immune cell infiltration in COAD. **(A)** Proportion of immune cells in COAD patients estimated by the CIBERSORT algorithm in TCGA data. **(B)** Differences in the level of immune cell infiltration between COAD and controls and between high-group and low-group. Red is high infiltration and blue is low infiltration. **(C)** Radar chart showing the correlation between DLL3 and immune cells in COAD. **(D)** Differential expression of DLL3 between COAD and control in TCGA, GSE39582 and GSE44076 data. ***p < 0.001. **(E)** ROC curves of DLL3 in TCGA, GSE39582 and GSE44076 datasets predicting COAD.

By assessing the proportion of immune cell types in the COAD samples, we found the maximum proportion of naive CD4^+^ T cells and M2 macrophage (Figure 6A). Differences in immune cell infiltration between COAD and control samples and between DLL3 high- and low-expression group (Figure 6B). The correlation between DLL3 and immune cells illustrates the highest correlation between DLL3 and iDCs, NK cells, and Tem (Figure 6C). The differential results showed significantly higher expression levels of DLL3 in COAD patients compared to the control group (Figure 6D). The ROC curves revealed that the AUC values of DLL3 in TCGA, GSE39582 and GSE44076 datasets for predicting COAD were all greater than 0.63 (Figure 6E).

### 3.7 Association of DLL3 with clinical features and overall survival in COAD

**FIGURE 7:**
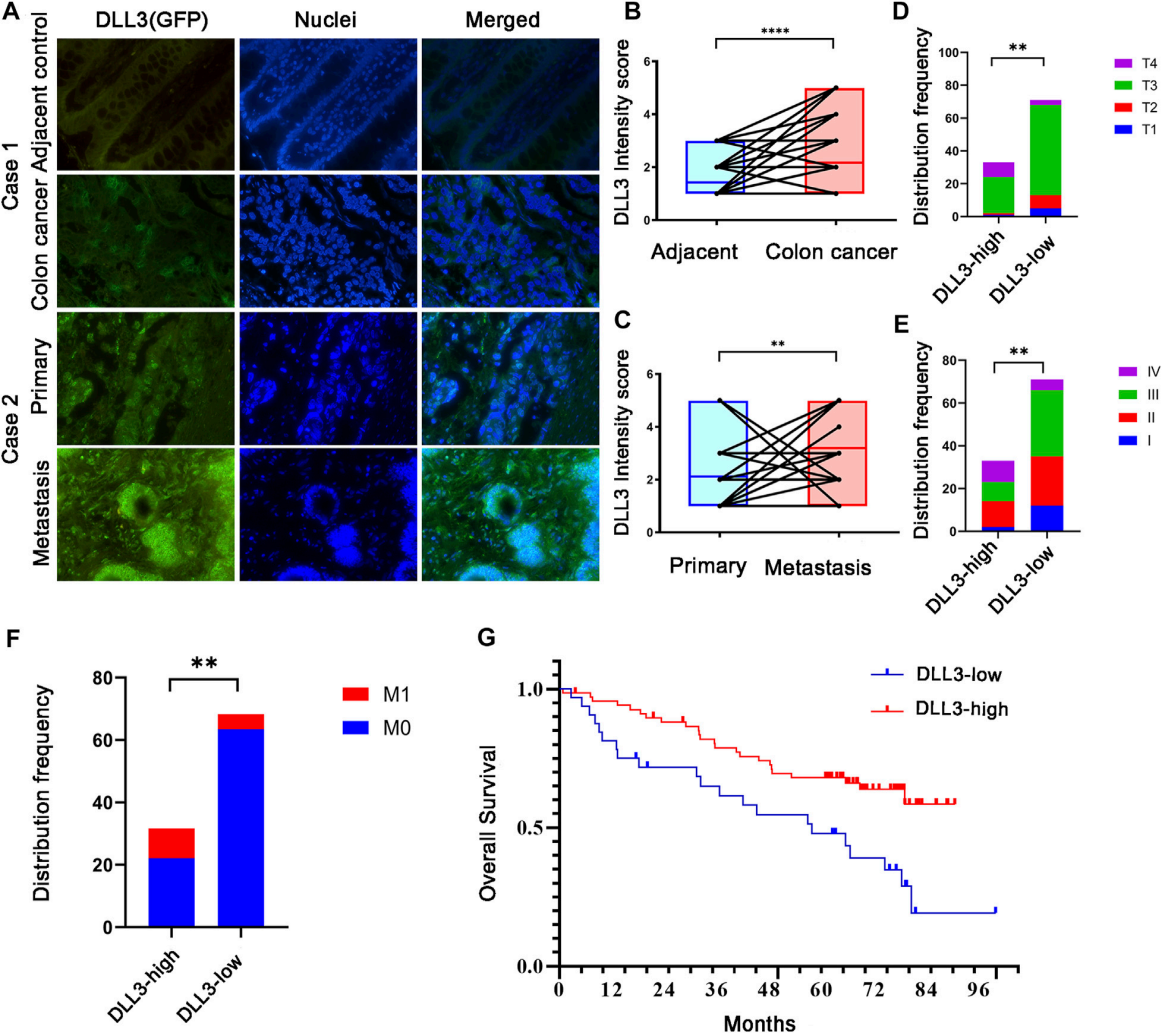
Expression and survival analysis of DLL3**. (A)** IHC staining of DLL3 in COAD samples (×400 magnification). Case 1: DLL3 expression in adjecent and tumor tissues. Case 2: DLL3 expression primary and metastatic lesion. **(B)** Paired analysis of DLL3 expression in normal and tumor tissues. **(C)** Paired analysis of DLL3 expression in cancer and metastatic lesion. **(D)** Disease stage and DLL3 **(E)** T stage and DLL3 **(F)** M stage and DLL3 **(G)** Kaplan–Meier survival analysis of COAD patients with OS based on DLL3 expression.

Tumor tissues and corresponding adjacent non-tumor tissues collected from 104 COAD patients were analyzed for DLL3 expression by immunofluorescence and DLL3 expression was also found to be higher in tumor tissues than in adjacent tissues (*p* < 0.0001, Figures 7A, B case1). We collected specimens from both primary and metastatic foci in 24 of these patients, and immunofluorescence staining revealed higher DLL3 expression in the metastases than in the primary lesion (*p* = 0.0056, Figures 7A–C case2). A comparison of the clinical characteristics of those with high and low DLL3 expression showed that disease stage (*p* = 0.0071, Figure 7D), T stage (*p* = 0.0044, Figure 7E), and M stage (*p* = 0.0049, Figure 7F) were associated with DLL3 expression. The expression of DLL3 wasn’t correlated with the location, metastatic organ and N stage (*p* > 0.05). Furthermore, it was found that high DLL3 expression was associated with poorer overall survival in patients with COAD (Log-rank *p* = 0.004, HR2.304, 95% CI of ratio 1.361–5.051; Figure 7G), which is indeed consistent with the results of the previous analysis of data.

## 4 Discussion

Our study integrated information from databases and found poorer 5-year survival of COAD patients in the DLL3 high compared to low-expression group. And this was subsequently verified in clinical samples. Enrichment analysis revealed that DLL3-related DEGs were mainly involved in tumor- and immune-associated signaling pathway, suggesting that DLL3 is significantly involved in the tumor development process. The KEGG pathways inhibited in the DLL3 high-expression group and COAD tumor were identified by GSEA: mitophagy-animal and AMPK pathway. The intersection genes were eventually screened for 10 signature genes, and a nomogram was further constructed to provide a promising prediction of COAD prognosis. We also found the highest correlation between DLL3 and three immune cells, iDC, NK cells and Tem, which may be useful for immunotherapy of COAD. DLL3 was also found to be expressed at significantly higher levels in COAD patients than in healthy controls in the database, suggesting a diagnostic function of DLL3 for colon carcinogenesis.

The original study from GSE39582 dataset (Marisa et al., 2013) aimed to establish a reliable molecular typing of COAD mRNA expression profiles and to validate six molecular subtypes, illustrating the biological relevance of these subtypes through significant differences in prognosis. The limitation of their study was that they didn't obtain information about tumor grade and number of lymph nodes examined. In our report, however, this was included and analyzed. Subtypes in the initial study were related to different clinicopathological features, molecular alterations, specific enrichments of supervised gene expression tags, and dysregulation of signaling pathways. Instead, we examined DLL3 and its related genes which affect the prognosis of COAD. The original study of GSE44076 (Sole et al., 2014) aimed to identify and validate new serum biomarkers and demonstrate their role in the potential early diagnosis of colon cancer. Eventually the process identifying serum levels of COL10A1 protein as a potential diagnostic candidate for the detection of adenomatous lesion and tumor. Unfortunately, DLL3 was not screened at that time, since study was comparing the difference in serum protein level between control, patients with adenoma and colon cancer cases. In contrast, our analysis compared COAD with healthy control and didn’t include the sample of colon adenoma. Unlike the related studies of the above two datasets, the innovation of our study is to screen for DLL3 and its related genes affecting the prognosis of COAD, identify the related functions and pathways, and construct a prognostic model for COAD.

Notably, overexpression of DLL3 was associated with shorter OS in endometrial cancer, ovarian cancer, breast cancer and Small-cell Bladder Cancer. That is consistent with the results obtained in this study of DLL3 in COAD. So it follows that DLL3 has a critical role in maintaining malignant growth and is associated with poor prognosis, particularly in relatively rare neuroendocrine subtypes. Whether DLL3 could be a potential therapeutic target for these tumors needs to be further investigated. Because DLL3 is overexpressed in certain tumor cells, the current principle of therapeutic targeting of DLL3 is mainly to kill cancer cells by targeting DLL3 with antibody-coupled cytotoxic drugs or by specifically targeting cancer cells with high DLL3 expression through CD3-activated T cells (Owen et al., 2019). Since our study revealed that DLL3 predicts poorer survival in COAD, the prognosis of COAD can be improved by targeting the inhibition of DLL3 expression. A previous microarray analysis (Meng et al., 2009) showed that DLL3 expression did not increase with colon cancer progression, but the sample size in this study was small and it was more than a decade ago. Although there was no significant difference in DLL3 at that time, DLL3 still tends to increase with colon cancer progression looking at the graph (Meng et al., 2009). Since most of the signaling pathways enriched by KEGG in this study were tumor- and immune-related, we conclude that DLL3 is involved in the development and progression of COAD. The classical Notch signaling pathway is involved in the maintenance of stem and progenitor cells in the colon, as well as in the inhibition of cupped cell differentiation (Sikandar et al., 2010; Prasetyanti et al., 2013). DLL3 inhibits the activation of Notch signaling pathway by interacting with Notch receptors. It can also bind to DLL1 and Notch1 receptor to inhibit modification and promote Notch1 receptor degradation, thus achieving inhibition of Notch signaling pathway activation as well as upregulation of expression (Owen et al., 2019). It is suggested that in addition to NOTCH pathway, DLL3 also plays a certain role in the development of COAD through other pathways. For instance, we found that DLL3-related intersection genes are associated with AMPK signaling pathway, cell cycle, mTOR signaling pathway, mitophagy−animal, etc.

Recent studies have shown that an ancestral function of AMPK is to promote mitochondrial health, and that multiple newly identified AMPK targets are involved in various aspects of mitochondrial homeostasis, which includes mitophagy (Herzig and Shaw, 2018). Autophagy is a specific cellular mechanism for recycling cellular components such as proteins, macromolecules, organelles and pathogens, which mediates the engulfment of material in autophagic vesicles by membrane structures, fusion with lysosomes and subsequent degradation of material in autophagic vesicles (Bento et al., 2016). AMPK has been proved to both regulate autophagy in yeast (Wang et al., 2001) and mammalian cells (Meley et al., 2006; Hoyer-Hansen et al., 2007). First, AMPK directly phosphorylates Thr1227 and Ser1345 of the mTOR upstream regulator TSC2 (Inoki et al., 2003) and Ser722 and Ser792 of the mTORC1 subunit RAPTOR (Gwinn et al., 2008). Both phosphorylation events are involved in reduction of mTOR activity under energetic stress conditions. The reduction in mTOR activity reduces the inhibitory phosphorylation of ULK1 to activate autophagy, as described above. At least three aspects of mitochondrial homeostasis are regulated by AMPK: biogenesis, fission, and mitophagy (Buck et al., 2016; Forni et al., 2016; Ho et al., 2017). Cell structure and function are maintained through mitophagy, one of the organelle-specific autophagy pathways (Okamoto, 2014; Onishi et al., 2021). Studies have confirmed that mitochondria-targeted drugs could stimulate mitophagy by activating AMPK signaling and abrogate colon cancer cell proliferation (Boyle et al., 2018). Under nutrient stress, colorectal cancer cells may also use mitophagy to maintain mitochondrial metabolism for proliferation (Devenport et al., 2021). Based on their findings, the dual nature of autophagy and mitophagy in cancer growth may be attributed to nutrient availability. In COAD, autophagy is known to promote tumor growth and suppress tumor (Mathew et al., 2007; Burada et al., 2015), but the underlying mechanism need to be explored further.

Based on the relationship between activation of AMPK signaling pathway and autophagy in previous studies, combined with the results of KEGG enrichment analysis of the intersection genes of DLL3 that affect the prognosis of COAD in the database, we found that the intersection genes are involved in the pathway with both AMPK signaling pathway, mitophagy-animal, and other tumor-related and immune-related pathways. The GSEA analysis then showed that both mitophagy-animal and AMPK pathways were suppressed in the DLL3 high-expression group of COAD. This is in accordance with several previous reports (Ziegler et al., 2018; Islam Khan and Law, 2021) on the state of mitophagy in COAD. Therefore, we need further basic experimental validation regarding the mechanism of DLL3’s action in COAD.

Of course, there are some limitations in our study. Firstly, because of limited specimens, we only validated the expression of DLL3 in clinical samples, but not other related genes. Secondly, the key role of DLL3 in COAD requires further *in vivo*/*in vitro* basic experiments to confirm, which is our next step.

## 5 Conclusion

In conclusion, DLL3 is highly expressed in human COAD tissues and is associated with poorer overall survival. The identification of DLL3-related genes suggests that DLL3 may affect COAD development by regulating cellular functions and pathways. DLL3 may be a target and prognostic marker for individualized treatment of COAD, and it may has a diagnostic role in colon carcinogenesis.

## Data Availability

The original contributions presented in the study are included in the article/Supplementary Material, further inquiries can be directed to the corresponding authors.

## Supplementary material

SUPPLEMENTARY FIGURE S1 DLL3-NOTCH pathway. The figure was prepared utilizing templates obtained from www.proteinlounge.com. NICD, NOTCH intracellular domain; CSL, CBF1/Suppressor of Hairless/LAG-1.

SUPPLEMENTARY FIGURE S2 The flow diagram of our study. GSEA, Gene Set Enrichment Analysis; LASSO, least absolute shrinkage and selection operator.

## Abbreviations

DLL3, delta-like ligand 3; TCGA, the Cancer Genome Altas; GEO, gene Expression Omnibus; COAD, colon adenocarcinoma; DEGs, differentially expressed genes; BP, biological process; GO, gene Ontology; CC, cellular component; MF, molecular function; GSEA, gene set enrichment analysis; iDC, interstitial dendritic cell, NK, natural killer cell; Tem, interstitial dendritic cell; DSL, delta/Serrate/Lag2; SCLC, small-cell lung cancer; Rova-T, Rovalpituzumab tesirine; GO, gene Ontology; KEGG, kyoto Encyclopedia of Genes and Genomes; K-M, kaplan–Meier; AUC, area Under Curve; LASSO, least Absolute Shrinkage and Selection Operator; CIBERSORT, cell type Identification by Estimating Relative Subsets of RNA Transcripts; BP, biological processes; CC, cell composition; MF, molecular functions.
