# Supplementary material for: Identification of DLL3-related genes affecting the prognosis of patients with colon adenocarcinoma
Source: Front Genet. 2023 May 18;14:1098190. doi: 10.3389/fgene.2023.1098190 (PMC10233108; doi:10.3389/fgene.2023.1098190)
Supplement: Supplementary file 1 [file Table1.DOCX]

| Variable | | DLL3-high | DLL3-low |  |
| --- | --- | --- | --- | --- |
| Total(n=104) | | 33 | 71 |  |
| Age | |  | | P=0.1451 |
|  | < 65 | 14 | 41 |  |
|  | ≥ 65 | 19 | 30 |  |
| Sex | |  | | P=0.0422 |
|  | Male | 25 | 39 |  |
|  | Female | 8 | 32 |  |
| Location | |  |  | P=0.7856 |
|  | Left colon | 20 | 45 |  |
|  | Right colon | 13 | 26 |  |
| Metastasis | |  |  | P=0.3304 |
|  | Liver | 8 | 13 |  |
|  | Lung | 3 | 10 |  |
|  | Others | 15 | 17 |  |
| Disease stage | |  |  | P=0.0071 |
|  | I | 2 | 12 |  |
|  | II | 12 | 23 |  |
|  | III | 9 | 31 |  |
|  | IV | 10 | 5 |  |
| T stage |  |  |  | P=0.0044 |
|  | T1 | 1 | 5 |  |
|  | T2 | 1 | 8 |  |
|  | T3 | 22 | 55 |  |
|  | T4 | 9 | 3 |  |
| N stage |  |  |  | P=0.3577 |
|  | N0 | 16 | 34 |  |
|  | N1 | 8 | 25 |  |
|  | N2 | 9 | 12 |  |
| M stage |  |  |  | P=0.0049 |
|  | M0 | 23 | 66 |  |
|  | M1 | 10 | 5 |  |
| Previous treatment |  |  |  | P=0.587 |
|  | Neoadjuvant | 0 | 0 |  |
|  | surgery | 18 | 40 |  |
|  | Adjuvant chemotherapy | 10 | 28 |  |
|  | Radiotherapy | 0 | 3 |  |
| MSI |  |  |  | P=0.773 |
|  | dMMR | 2 | 8 |  |
|  | pMMR | 12 | 23 |  |
|  | Unknown | 4 | 9 |  |

*Supplement Table 1.* *The characteristics*
