# Supplementary figures and images for: Identification of DLL3-related genes affecting the prognosis of patients with colon adenocarcinoma
Source: Front Genet. 2023 May 18;14:1098190. doi: 10.3389/fgene.2023.1098190 (PMC10233108; doi:10.3389/fgene.2023.1098190)

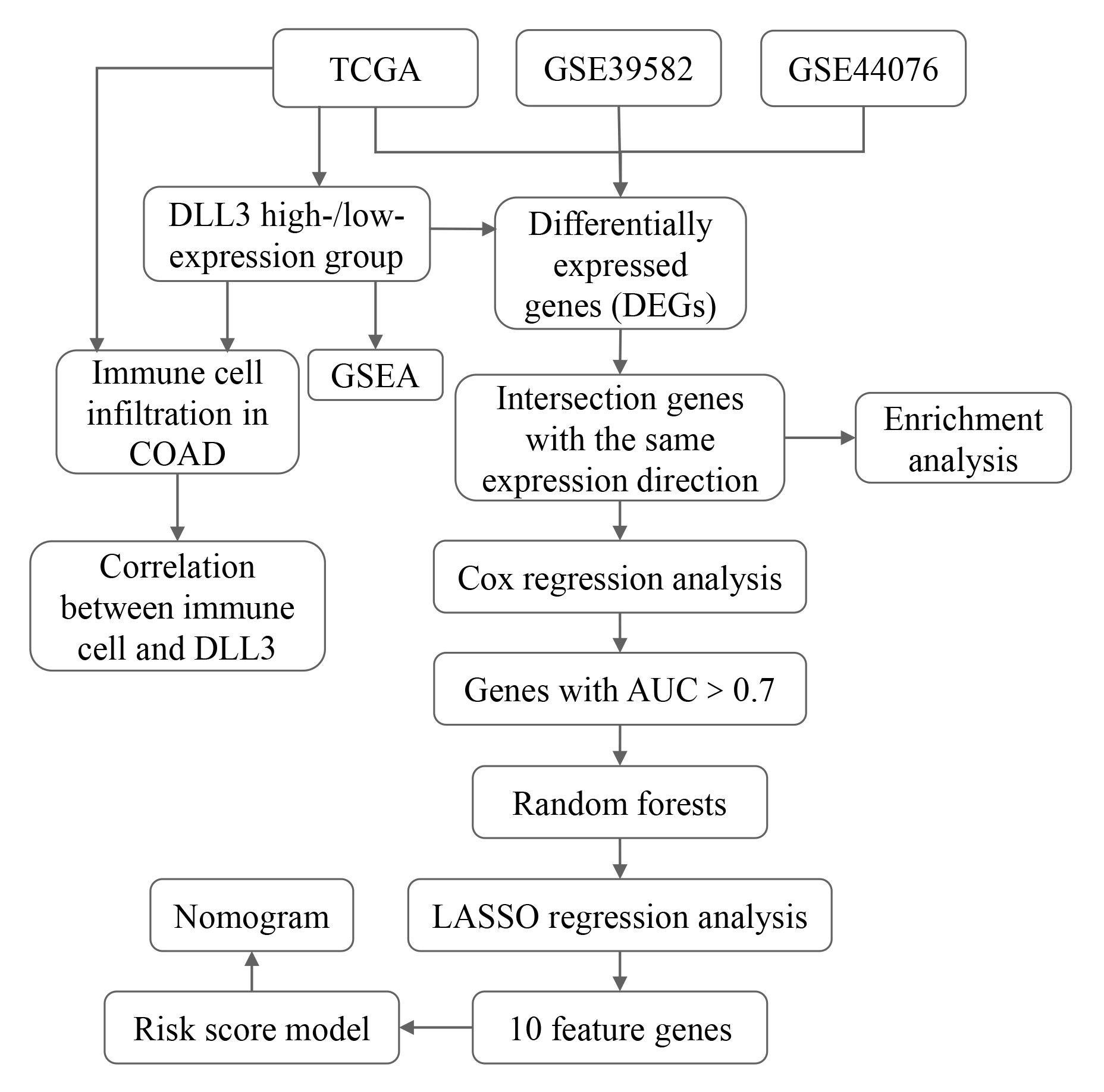

Supplement: Supplementary file 2 [file Image1.TIF]
